# Supplementary material for: Contemporary HIV-1 consensus Env with AI-assisted redesigned hypervariable loops promote antibody binding
Source: Nat Commun. 2024 May 9;15:3924. doi: 10.1038/s41467-024-48139-x (PMC11082178; doi:10.1038/s41467-024-48139-x)
Supplement: Supplementary file 1 — Supplementary Information [file 41467_2024_48139_MOESM1_ESM.pdf]

## Supplementary material

### Table S1. Summary measures for V1, V2, V4 and V5 HV loop lengths.

| | V1HV | | | V2HV | | | V4HV | | | V5HV | | |
|---|---|---|---|---|---|---|---|---|---|---|---|---|
| | B | C | 01_AE | B | C | 01_AE | B | C | 01_AE | B | C | 01_AE |
| count | 2495 | 1503 | 849 | 2495 | 1503 | 849 | 2495 | 1503 | 849 | 2495 | 1503 | 849 |
| mean | 22.8 | 20.0 | 23.8 | 8.0 | 9.1 | 8.5 | 12.5 | 9.2 | 8.6 | 9.2 | 9.2 | 8.5 |
| std | 6.0 | 6.1 | 4.1 | 3.7 | 3.5 | 3.6 | 3.7 | 4.4 | 3.5 | 2.1 | 2.4 | 1.8 |
| min | 3 | 5 | 7 | 0 | 0 | 0 | 0 | 0 | 0 | 0 | 1 | 2 |
| 25% | 19 | 16 | 22 | 6 | 6 | 6 | 11 | 6 | 7 | 8 | 8 | 7 |
| 50% | 22 | 20 | 23 | 7 | 9 | 8 | 12 | 10 | 9 | 9 | 9 | 8 |
| 75% | 26 | 24 | 26 | 10 | 11 | 10 | 15 | 12 | 11 | 10 | 10 | 9 |
| max | 44 | 40 | 40 | 26 | 23 | 27 | 27 | 24 | 22 | 19 | 21 | 17 |

### Table S2. Summary measures for the number of PNGS in V1, V2, V4 and V5 HV loops.

| | V1HV | | | V2HV | | | V4HV | | | V5HV | | |
|---|---|---|---|---|---|---|---|---|---|---|---|---|
| | B | C | 01_AE | B | C | 01_AE | B | C | 01_AE | B | C | 01_AE |
| count | 2495 | 1503 | 849 | 2495 | 1503 | 849 | 2495 | 1503 | 849 | 2495 | 1503 | 849 |
| mean | 2.6 | 2.6 | 3.1 | 1.2 | 1.2 | 1.1 | 2.4 | 2.0 | 1.8 | 1.5 | 1.3 | 1.6 |
| std | 1.0 | 1.0 | 1.0 | 0.6 | 0.6 | 0.6 | 0.9 | 1.0 | 0.7 | 0.6 | 0.6 | 0.5 |
| min | 0 | 0 | 0 | 0 | 0 | 0 | 0 | 0 | 0 | 0 | 0 | 0 |
| 25% | 2 | 2 | 3 | 1 | 1 | 1 | 2 | 1 | 1 | 1 | 1 | 1 |
| 50% | 3 | 2 | 3 | 1 | 1 | 1 | 2 | 2 | 2 | 2 | 1 | 2 |
| 75% | 3 | 3 | 4 | 2 | 2 | 1 | 3 | 3 | 2 | 2 | 2 | 2 |
| max | 7 | 7 | 7 | 5 | 4 | 5 | 5 | 5 | 5 | 4 | 4 | 3 |

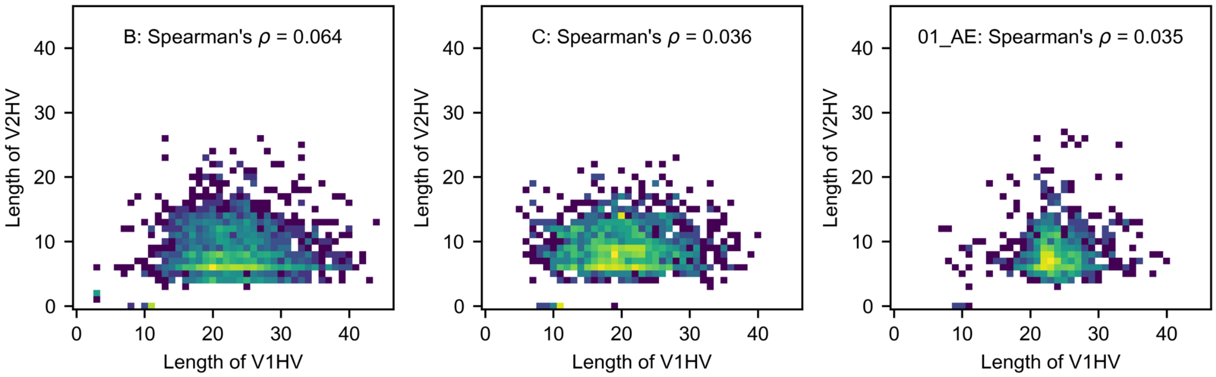


**Fig. S1. Lack of correlation between HIV-1 Env hypervariable loop lengths for V1HV and V2HV.** The distribution of loop lengths is shown for subtypes B (n=2495) and C (n=1503), and CRF01_AE (n=849) with Spearman's correlation coefficient labeled. The number of sequences of identical length are color-coded from dark blue (minimum number, n=1) to yellow (maximum number, n=92, 23, 29 for subtype B, C and CRF01_AE, respectively) after a log transformation. Source data for all panels are provided in the Source Data files.

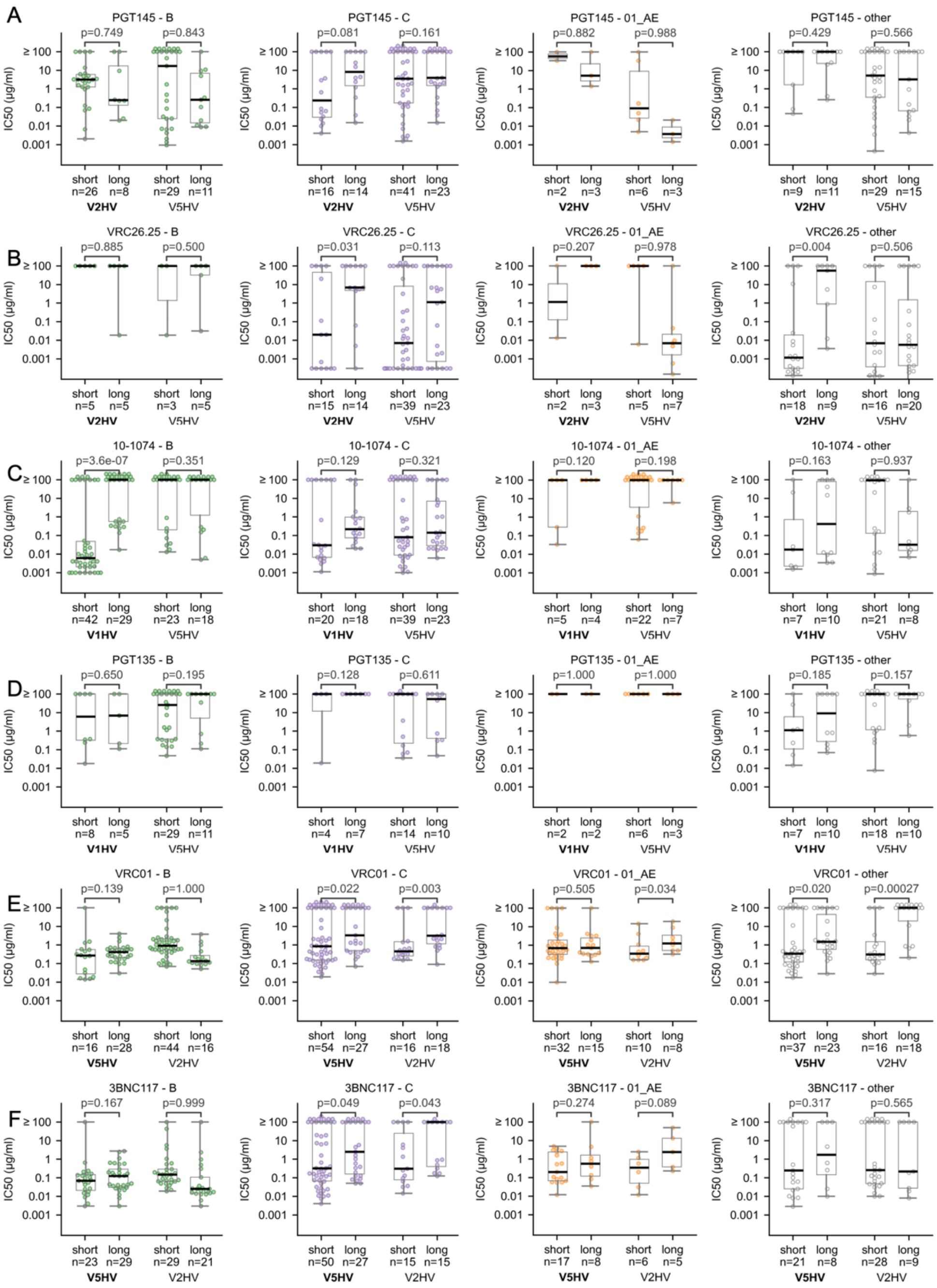

**Fig. S2. Comparison of the bnAb sensitivity for HIV-1 Env with short (≤ 5 percentile) or long (≥ 95 percentile) hypervariable (HV) loops for different subtypes.** Comparisons were performed on two V2 apex-targeting antibodies whose epitopes are close to V2HV loops: PGT145 (A) and VRC26.25 (B); two glycan supersite antibodies whose epitopes are close to V1HV loops: 10-1074 (C) and the PGT135 (D); and two CD4 binding site antibodies with epitopes close to the V5HV loops: VRC01 (E) and 3BNC117 (F). For each panel, comparisons of short and long HV loops are shown for the HV loop adjacent to (left) and distant from (right) the Ab epitope. Comparisons are shown for sequences corresponding to specific subtypes/CRF and for the set of sequences that were not subtype B, subtype C or CRF01_AE ('other'). The p-values correspond to non-paired, non-parametric Mann-Whitney U tests. The minimum, maximum, first

and third quartiles and median of the distribution are shown with box plots. IC50 values equal to or greater than 100 µg/ml are grouped (this corresponds to the limit for antibody neutralization measurements in the CATNAP database). Source data for all panels are provided in the Source Data files.

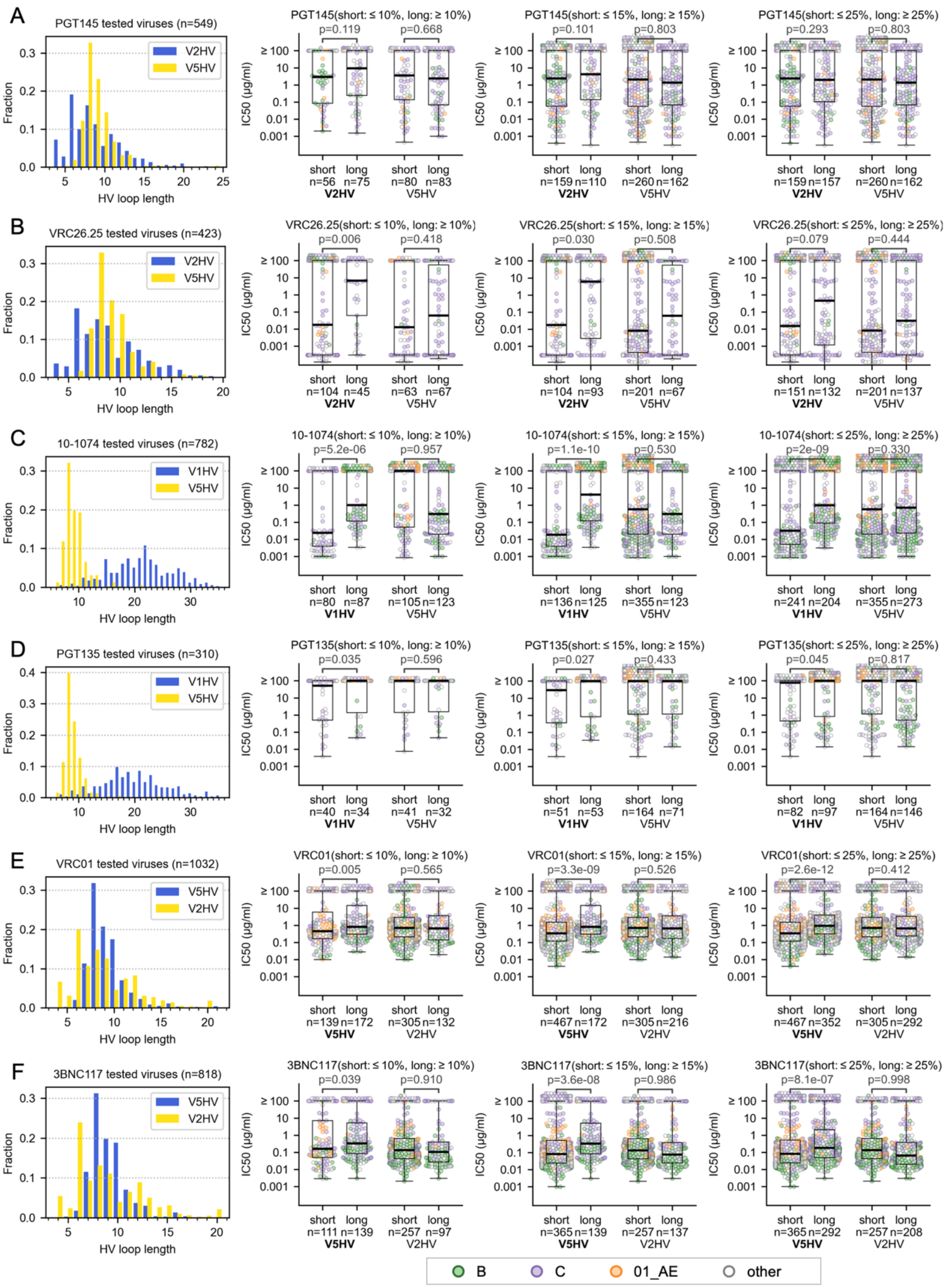

**Fig. S3. Comparison of the bnAb sensitivity for HIV-1 Env using different cutoffs for Env with the shortest or longest hypervariable (HV) loops.** Comparisons are based on six antibodies: PGT145 (A), VRC26.25 (B), 10-1074 (C), PGT135 (D), VRC01 (E) and 3BNC117 (F). The first column shows the distribution of HV loop lengths for tested viruses in CATNAP. The second to fourth columns are the comparisons of neutralization sensitivity values between viruses

with short and long HV loops (cutoff of 10%, 15% or 25%) for loops that are either adjacent or distant from the Ab epitope. The number of viruses can be the same for different cutoffs, as viruses of same HV loop length can correspond up to 40% of all the tested Env sequences (e.g. V5HV of PGT135 tested viruses). The p-values correspond to non-paired, non-parametric Mann-Whitney U tests. The minimum, maximum, first and third quartile, and the median of the distribution are shown with box plots**.** IC50 values equal to or greater than 100 μg/ml are grouped (this corresponds to the limit for antibody neutralization measurements in the CATNAP database). Source data for all panels are provided in the Source Data files.

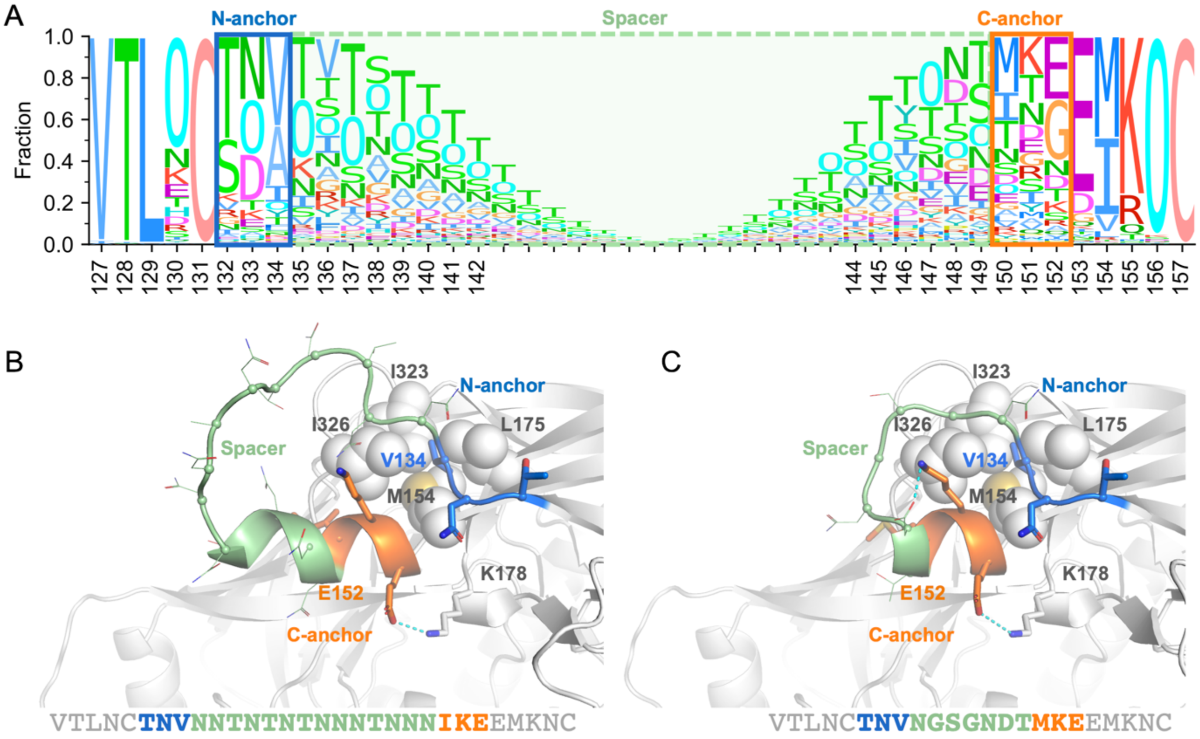


**Fig. S4. Redesigned V1 hypervariable loop of the subtype C consensus sequence.** (A) Sequence logo of 1503 subtype C sequences around the V1HV loop, with the N-/C-anchors and spacer indicated by boxes. The letter ‘O’ indicates a potential N-linked glycosylation site. The consensus subtype C Env with unmodified V1HV loop (B) and redesigned V1HV loop (C) modeled by AlphaFold2 are shown with the N-anchor, C-anchor and spacer sites colored blue, orange and light green, respectively. Non-HV residues that interact with anchor sites are shown as spheres (hydrophobic interaction) or sticks (charge-charge interactions). T132 and N133 in the N-anchor were chosen because of their prevalence in the sequence database. V134 was retained as it is the most frequent and it forms hydrophobic interactions with surrounding sites. Similarly, K151 in the C-anchor was chosen because of its sequence prevalence. M150 and E152 were retained as they form either hydrophobic interactions or charge-charge interactions with surrounding residues. Source data for panel (A) are provided in the Source Data file.

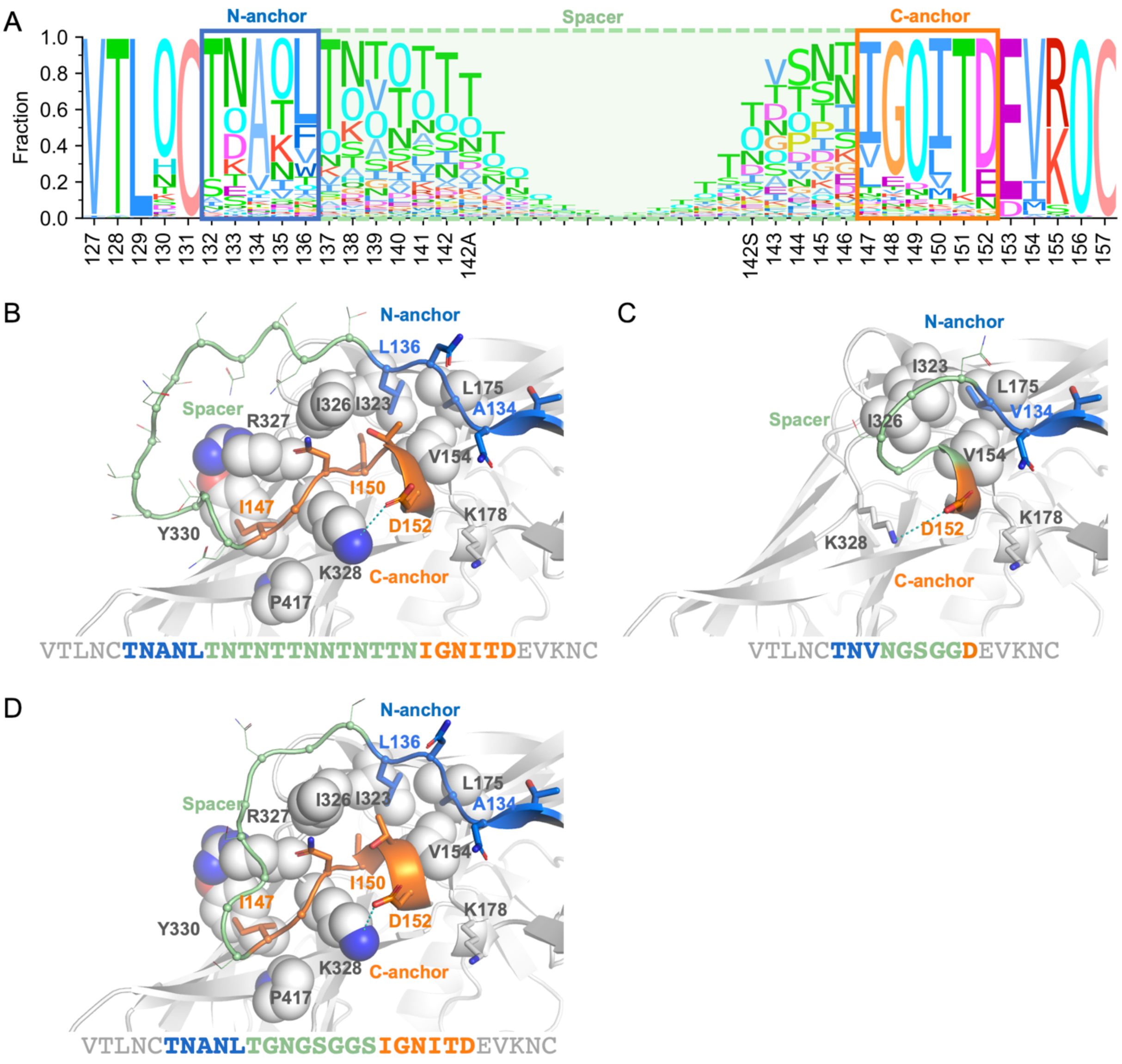


**Fig. S5. Redesigned V1 hypervariable loop of the CRF01_AE consensus sequence.** (A) Sequence logo of 849 CRF01_AE sequences around the V1HV loop, with the N-/C-anchors and spacer indicated by boxes. The letter ‘O’ indicates a potential N-linked glycosylation site. The consensus CRF01_AE Env with unmodified V1HV loop (B) and redesigned V1HV loop (C, D) modeled by AlphaFold2 are shown with the N-anchor, C-anchor and spacer sites colored blue, orange and light green, respectively. The conserved C-anchor sites ‘IGNITD’ are replaced by ‘D’ to improve antibody accessibility to the 10-1074 epitope (C) or kept as is in the expectation of better recognition by CRF01_AE antibodies (D). Non-HV residues that interact with anchor sites are shown as spheres (hydrophobic interactions) or sticks (charge-charge interactions). T132, N133 and N135 in the N-anchor were chosen because of their prevalence in the sequence database. A134 and L136 were retained because they are conserved and form hydrophobic interactions with surrounding sites. Similarly, G148, N149 and T151 in the C-anchor were chosen because of sequence conservation. I147, I150 and D152 were retained as they form either hydrophobic interactions or charge-charge interactions with surrounding residues. Source data for panel (A) are provided in the Source Data file.

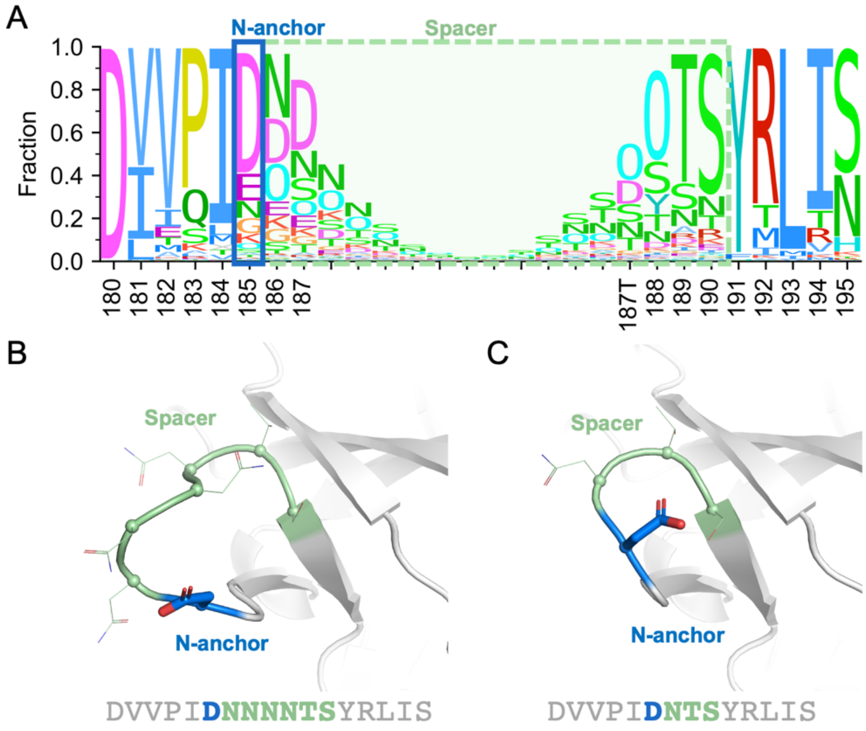


**Fig. S6. Redesigned V2 hypervariable loop of the subtype B consensus sequence.** (A) Sequence logo of 2495 subtype B sequences around the V2HV loop, with the N-anchor and spacer indicated by boxes. The letter 'O' indicates a potential N-linked glycosylation sites. The consensus subtype B Env with unmodified V2HV loop (B) and redesigned V2HV loop (C) modeled by AlphaFold2 are shown with the N-anchor and spacer sites colored blue and light green, respectively. D185 is considered as an anchor site because it is conserved and there is an enrichment of negatively charged residues (E and D) on the N-terminal of subtype B sequences. On the prefusion closed Env trimer, D185 is near the positively charged V2-apex and might promote the assembly of the Env trimer. It may also interact with S190 to stabilize the β-turn. No C-anchor site is assigned for the V2HV loop but redesigned HV loops are selected only if predicted LDDT scores after the C-terminal of the V2HV loop are similar or higher to the original Env when evaluating the AlpahFold2 modeled Env. Source data for panel (A) are provided in the Source Data file.

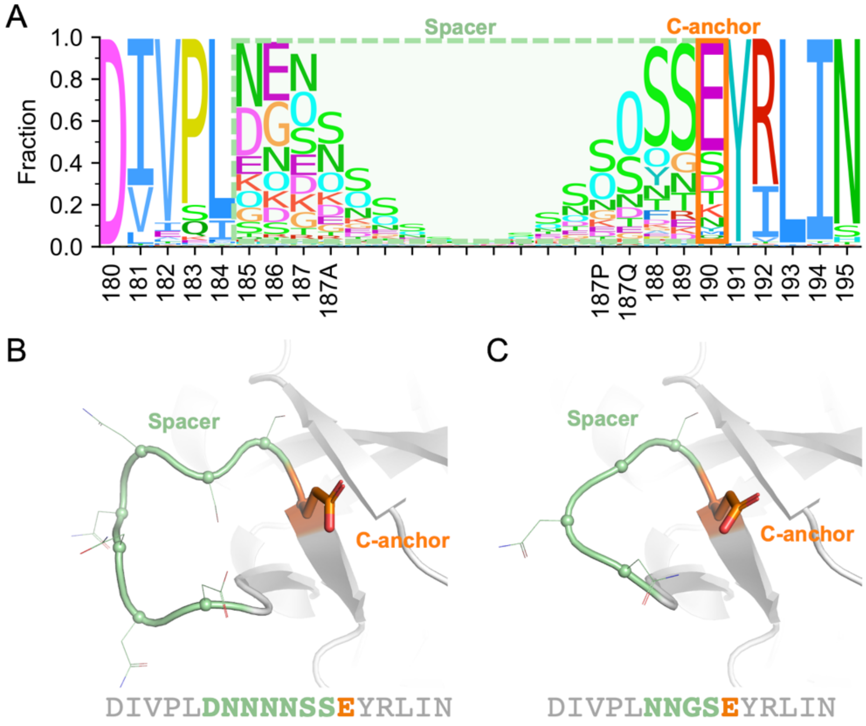


**Fig. S7. Redesigned V2 hypervariable loop of the subtype C consensus sequence.** (A) Sequence logo of 1503 subtype C sequences around the V2HV loop, with the C-anchors and spacer indicated by boxes. The letter 'O' indicates a potential N-linked glycosylation site. The consensus subtype C with unmodified V2HV loop (B) and redesigned V2HV loop (C) modeled by AlphaFold2 are shown with the C-anchor and spacer sites colored orange and light green, respectively. E190 is considered as an anchor site because it is relatively conserved and there is an enrichment of negatively charged residues (E and D) on the N/C-terminal of subtype C sequences. On the prefusion closed Env trimer, E190 is near the positively charged V2-apex and might promote the assembly of the Env trimer. No N-anchor site is assigned and the predicted LDDT score before the N-terminal of V2HV loop is selected to be similar or higher to the original Env when Env with redesigned HV loops are modeled and evaluated with AlpahFold2. Source data for panel (A) are provided in the Source Data file.

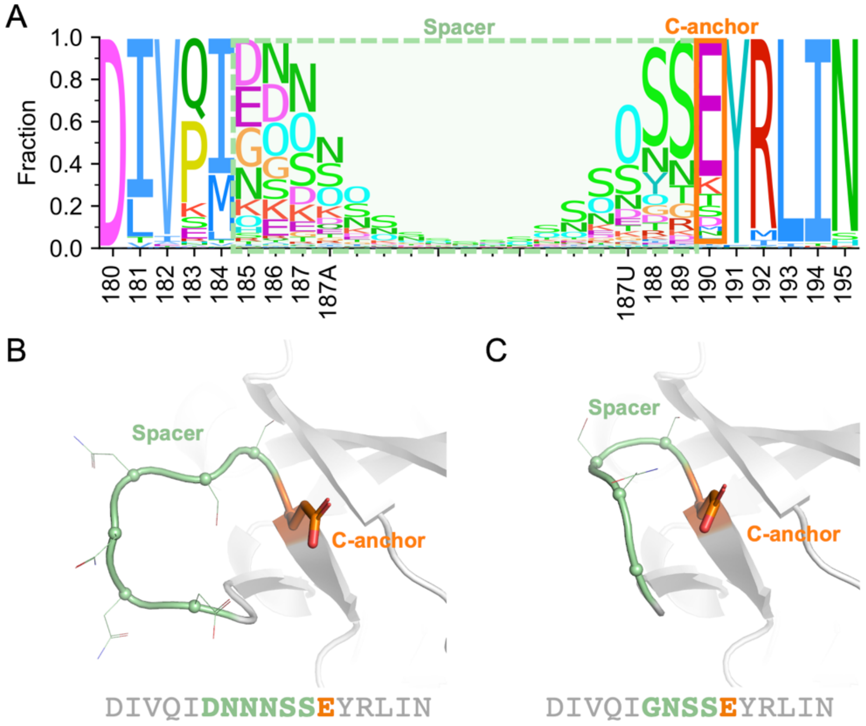


**Fig. S8. Redesigned V2 hypervariable loop of CRF01_AE consensus sequence.** (A) Sequence logo of 849 CRF01_AE sequences around the V2HV loop, with the C-anchors and spacer indicated by boxes. The letter 'O' indicates a potential N-linked glycosylation site. The consensus CRF01_AE Env with unmodified V2HV loop (B) and redesigned V2HV loop (C) modeled by AlphaFold2 are shown with the C-anchor and spacer sites colored orange and light green, respectively. E190 is considered as an anchor site because it is conserved and there is an enrichment of negatively charged residues (E and D) on the N/C-terminal of subtype C sequences. On the prefusion closed Env trimer, E190 is near the positively charged V2-apex and might assist in the assembly of the Env trimer. No N-anchor site is assigned and the predicted LDDT score before the N-terminal of V2HV loop is selected to be similar or higher to the original Env when Env with redesigned HV loops are modeled and evaluated with AlpahFold2. Source data for panel (A) are provided in the Source Data file.

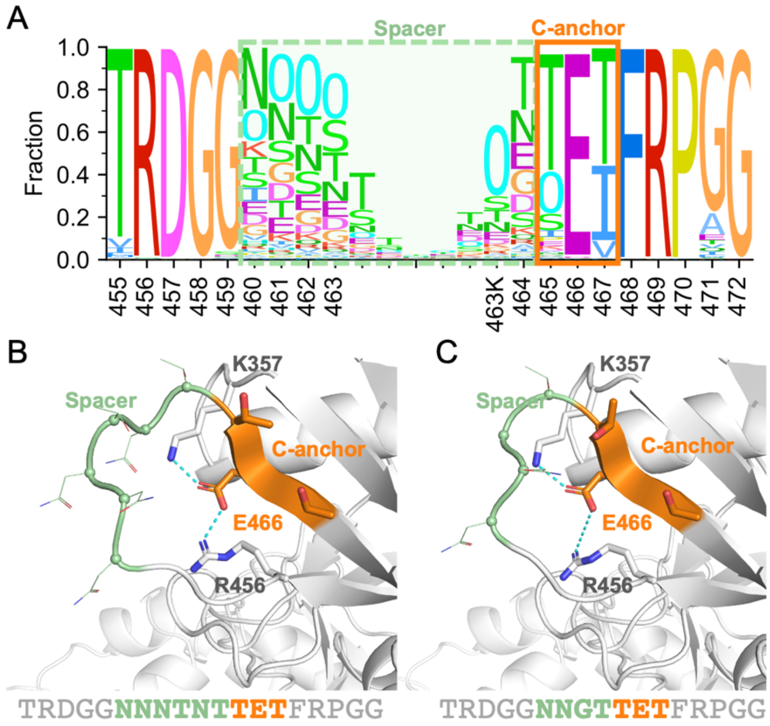


**Fig. S9. Redesigned V5 hypervariable loop of the subtype B consensus sequence.** (A) Sequence logo of 2495 subtype B sequences around the V5HV loop, with the C-anchor and spacer indicated by boxes. The letter ‘O’ indicates a potential N-linked glycosylation site. The consensus subtype B Env with unmodified V5HV loop (B) and redesigned V5HV loop (C) modeled by AlphaFold2 are shown with the C-anchor and spacer sites colored orange and light green, respectively. Non-HV residues that interact with anchor sites are shown as sticks. No N-anchor is identified as ‘GG’ before the N-terminal of V5HV is flexible and R456 and D457 are the nearest residues that have a well-defined conformation. In the C-anchor, T465 and T467 are selected as they are most the frequent residues, while E466 is conserved and forms charge-charge interactions with K357 and R456. Source data for panel (A) are provided in the Source Data file.

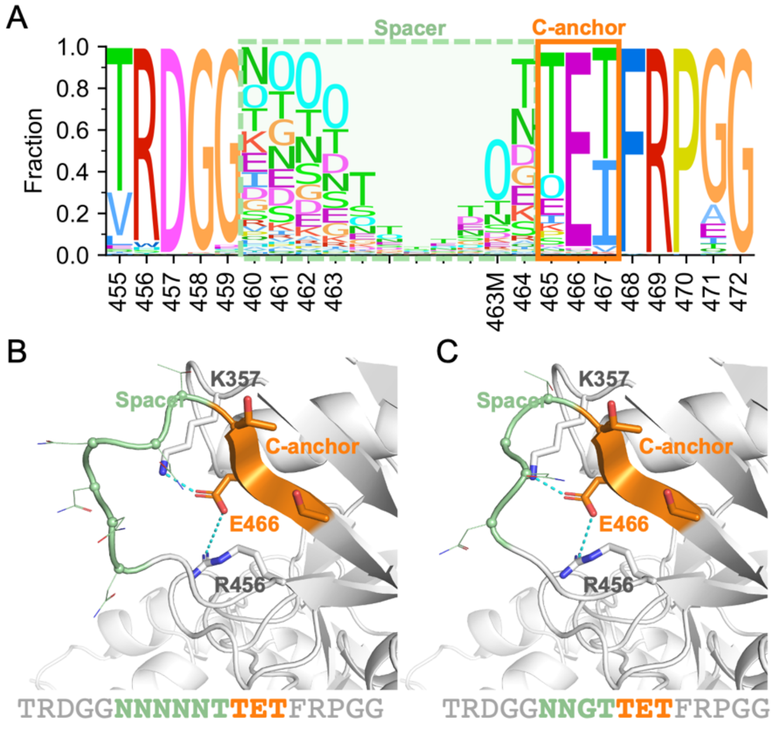


**Fig. S10. Redesigned V5 hypervariable loop of the subtype C consensus sequence.** (A) Sequence logo of 1503 subtype C sequences around the V5HV loop, with the C-anchor and spacer indicated by boxes. The letter 'O' indicates a potential N-linked glycosylation sites. The consensus subtype C Env with unmodified V5HV loop (B) and redesigned V5HV loop (C) modeled by AlphaFold2 are shown with the C-anchor and spacer sites colored orange and light green, respectively. Non-HV residues that interact with anchor sites are shown as sticks. No N-anchor is identified, as 'GG' before the N-terminal of V5HV is flexible and R456 and D457 are the nearest residues that have a well-defined conformation. In the C-anchor, T465 and T467 are selected as they are the most frequent residues, while E466 is conserved and forms charge-charge interactions with K357 and R456. Source data for panel (A) are provided in the Source Data file.

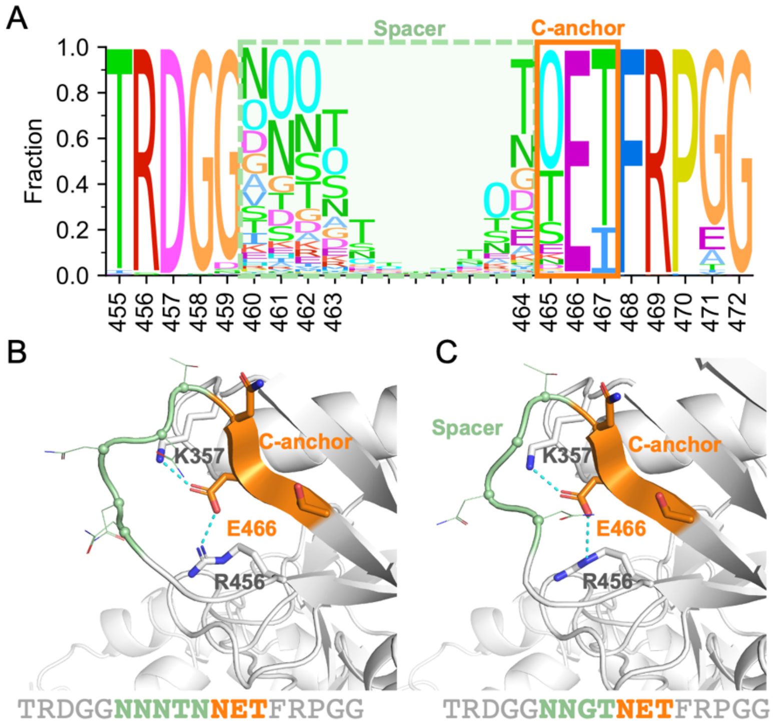


**Fig. S11. Redesigned V5 hypervariable loop of CRF01_AE consensus sequence.** (A) Sequence logo of 849 CRF01_AE sequences around the V5HV loop, with the C-anchor and spacer indicated by boxes. The letter 'O' indicates a potential N-linked glycosylation site. The consensus CRF01_AE Env with unmodified V5HV loop (B) and redesigned V5HV loop (C) modeled by AlphaFold2 are shown with the C-anchor and spacer sites colored orange and light green, respectively. Non-HV residues that interact with anchor sites are shown as sticks. No N-anchor identified, as 'GG' before the N-terminal of V5HV is flexible and R456 and D457 are the nearest residues that have a well-defined conformation. In the C-anchor, N465 and T467 are selected as they are the most frequent residues, while E466 is conserved and forms charge-charge interactions with K357 and R456. Source data for panel (A) are provided in the Source Data file.

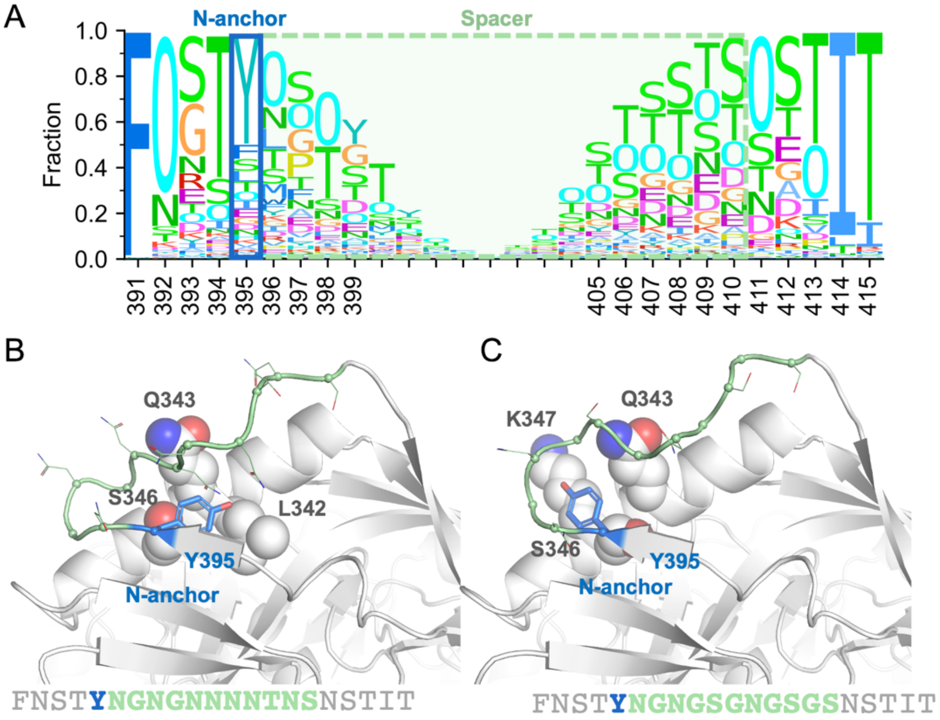


**Fig. S12. Redesigned V4 hypervariable loop of the subtype C consensus sequence.** (A) Sequence logo of 1503 subtype C sequences around the V4HV loop, with the N-anchors and spacer indicated by boxes. The letter 'O' indicates a potential N-linked glycosylation site. The consensus subtype C Env with unmodified V4HV loop (B) and redesigned V4HV loop (C) modeled by AlphaFold2 are shown with the N-anchor and spacer sites colored blue and light green, respectively. Non-HV residues that interact with anchor sites are shown as spheres. Y395 is considered as the N-anchor because it might form hydrophobic interactions with surrounding non-HV sites. No C-anchor is assigned because the C-terminal sites of V4HV loop are diverse and form no notable interactions with other sites. Source data for panel (A) are provided in the Source Data file.

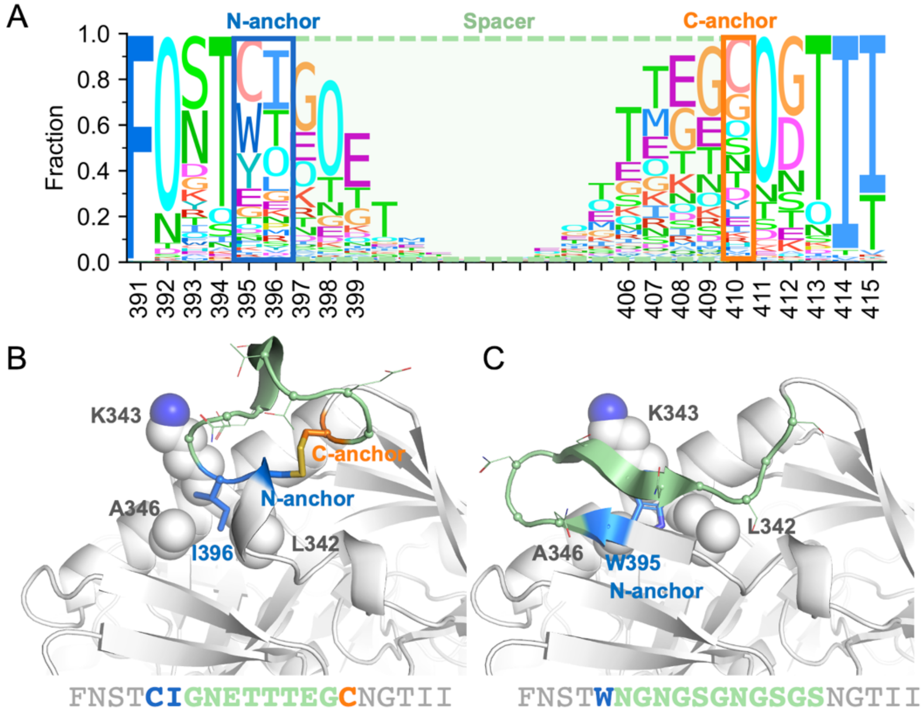


**Fig. S13. Redesigned V4 hypervariable loop of CRF01_AE consensus sequence.** (A) Sequence logo of 849 CRF01_AE sequences around the V4HV loop, with the N-/C-anchors and spacer indicated by boxes. The letter 'O' indicates a potential N-linked glycosylation site. The consensus CRF01_AE Env with unmodified V4HV loop (B) and redesigned V4HV loop (C) modeled by AlphaFold2 are shown with the N-anchor, C-anchor and spacer sites colored blue, orange and light green, respectively. Non-HV residues that interact with anchor sites are shown as spheres. In the predicted structure of the unmodified CRF01_AE consensus sequence, C395 and C410 form a disulfide bond and I396 forms hydrophobic interactions with non-HV sites, resulting in a constrained conformation for V4HV. Compared to other subtypes, CRF01_AE sequences have a shorter V4HV. Overall, the surrounding area of V4HV is better exposed in CRF01_AE sequences. In the redesigned sequence, we replaced C395 with the second most frequent residue, W, and extended the spacer to better shield the surrounding sites of V4HV. Source data for panel (A) are provided in the Source Data file.

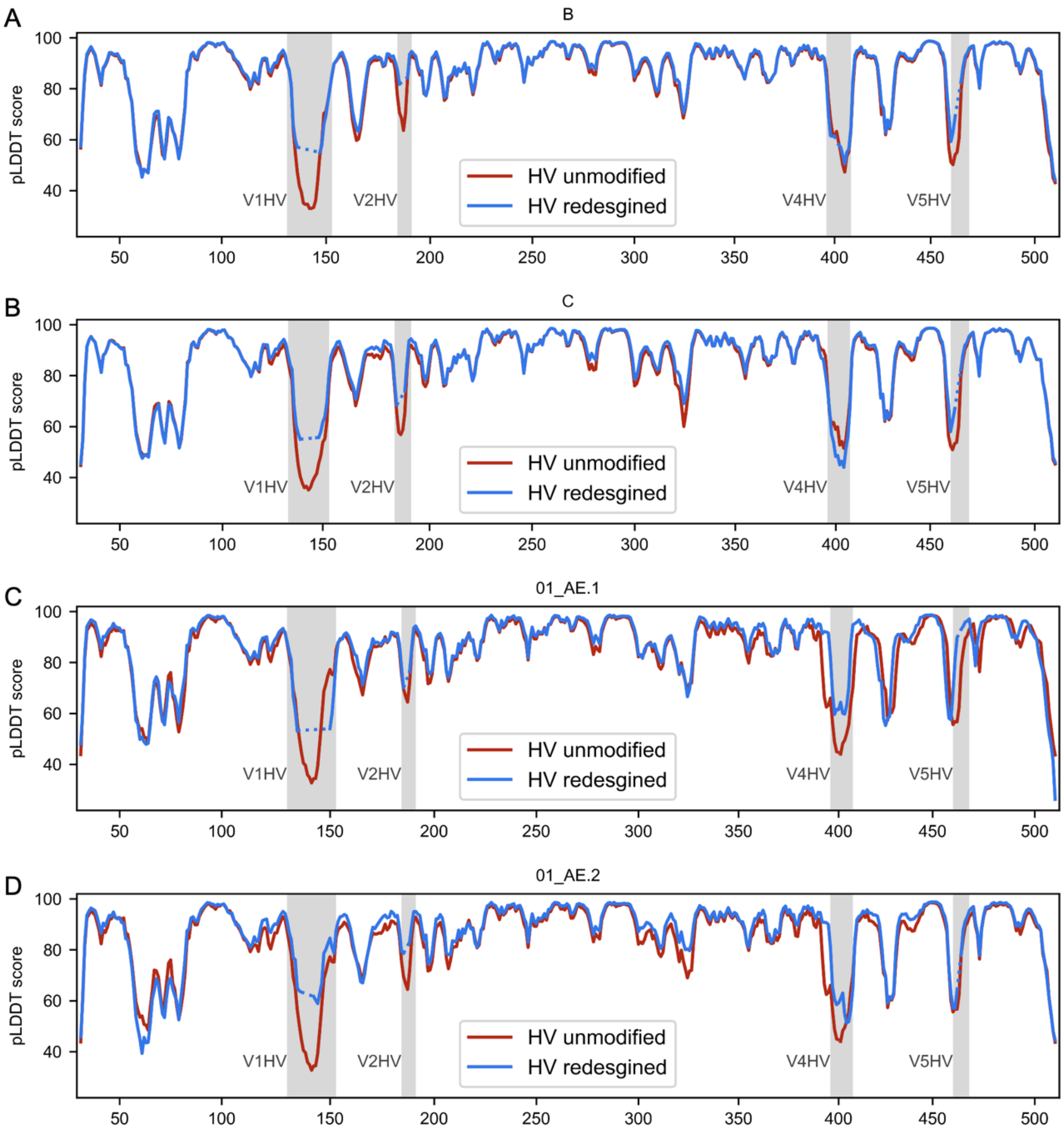


**Fig. S14. Comparison of the predicted local Distance Difference Test (lDDT) scores between HV unmodified and HV redesigned Env models.** The plDDT score is shown for Env with or without redesigned HV loop models for subtypes B (A) and C (B) and CRF01_AE (C and D). The x-axis is the HXB2 numbered positions of gp120. HV loops are highlighted with a gray shade. Source data are provided in the Source Data files.

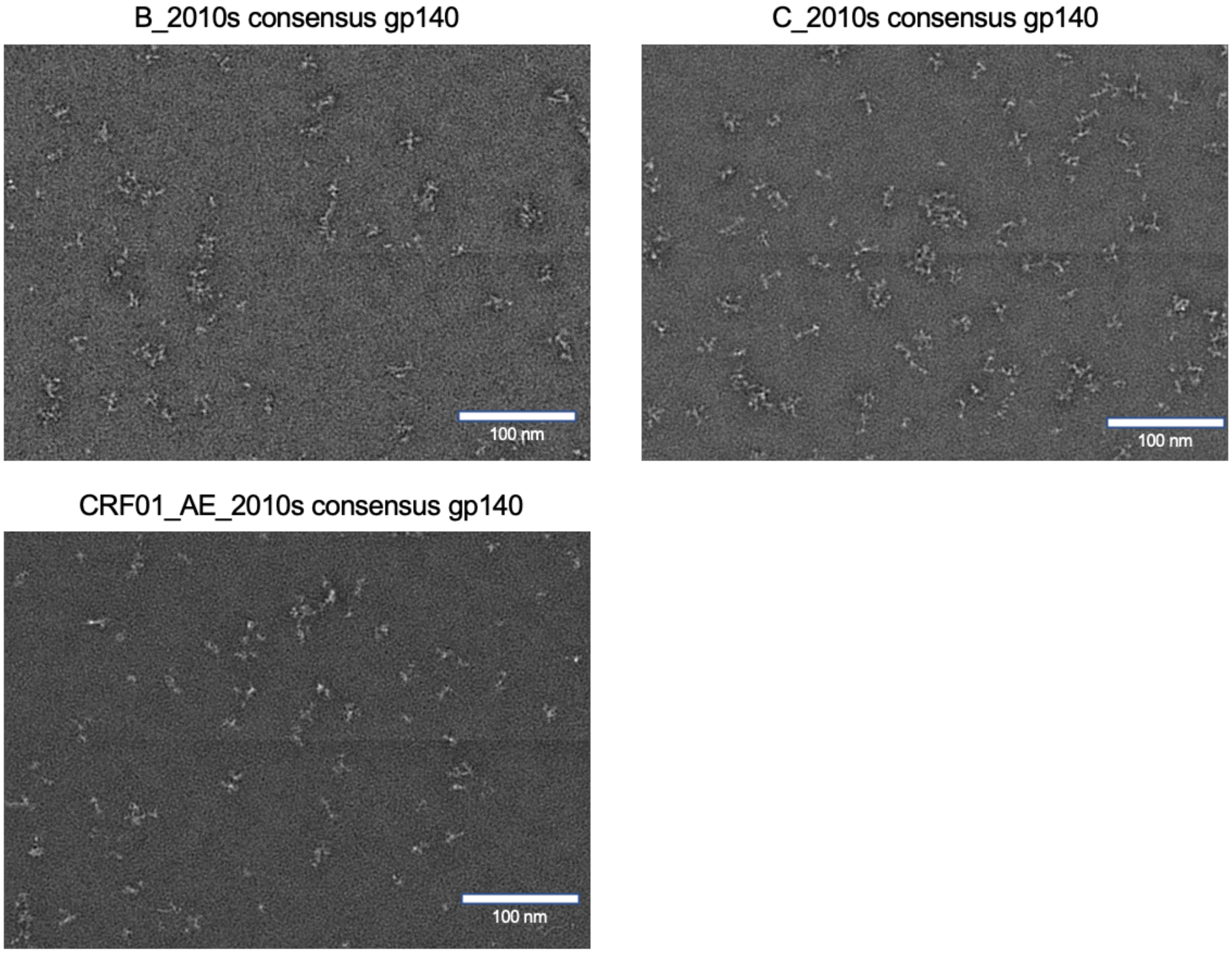


**Fig. S15. Negative-stain electron micrographs of HIV-1 Env gp140s.** Negative-Stain micrographs of gp140s without HV loop redesign showed almost entirely open trimers and only monomeric classes were averageable. For clarity, micrographs were lowpass filtered and binned (real space) using EMAN2 to enhance contrast.

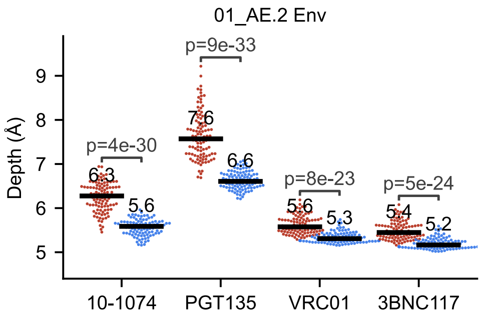


**Fig. S16. Antibody accessibility for four representative antibodies before and after the HV loop redesign for the variant CRF01_AE.2 (01_AE.2).** The definition of depth is illustrated in Figure 5. The p-value from non-paired, non-parametric one-tailed Mann-Whitney U tests is indicated (n=100 modeled conformations). Source data are provided in the Source Data file.

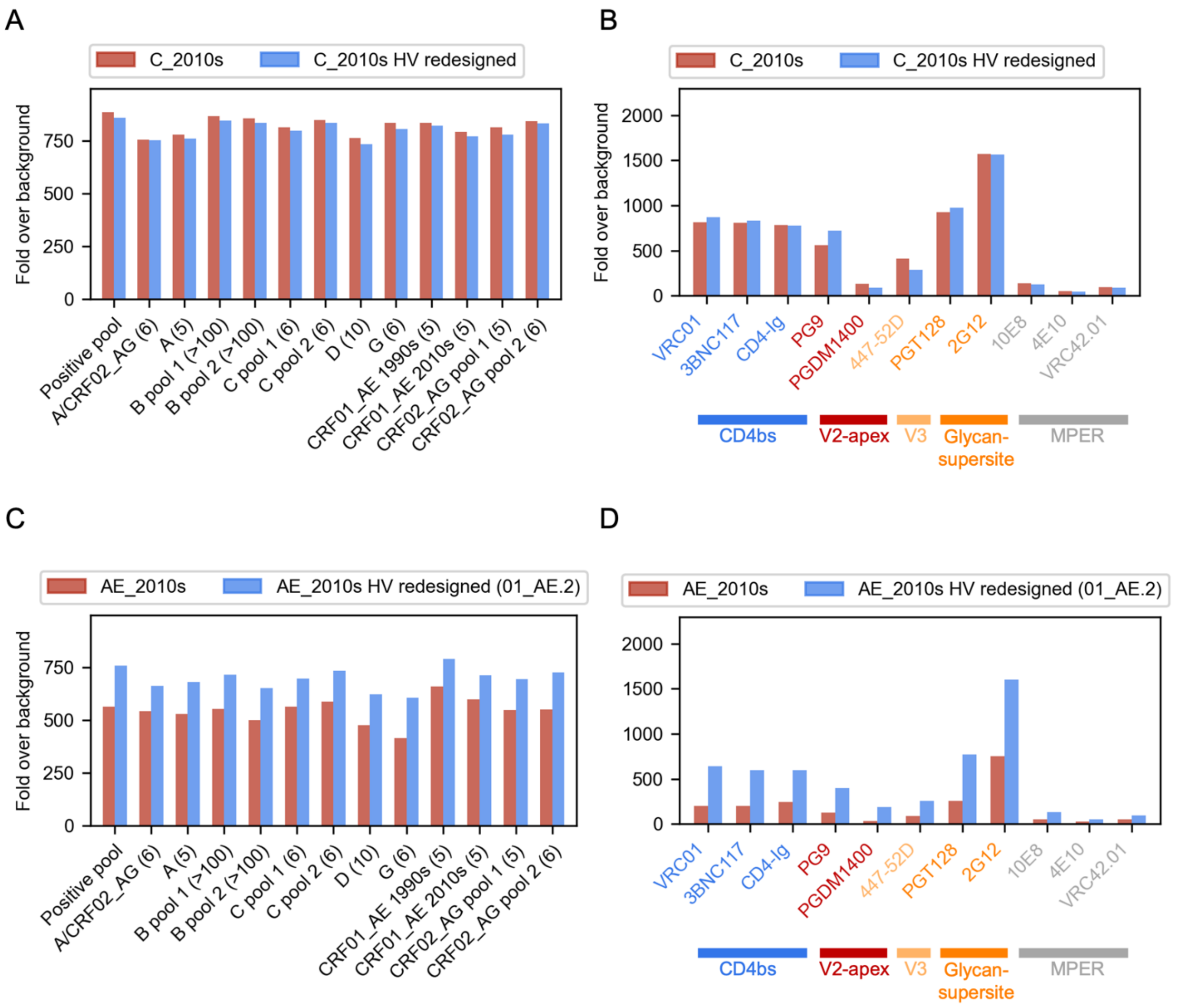


**Fig. S17. Antibody binding to redesigned consensus Env from subtype C and CRF01_AE.** Consensus Env sequences with unmodified and redesigned HV loops were expressed as gp140 proteins to compare binding to 13 plasma pools combined from different cohorts of PLWH (A, C) and to monoclonal antibodies (B, D). The number of plasma samples in each pool is shown in parenthesis in panel A/C. The consensus C and CRF01_AE Envs with redesigned HV loops are colored in blue and Envs with unmodified HV loops are colored in red. Source data are provided in the Source Data files.

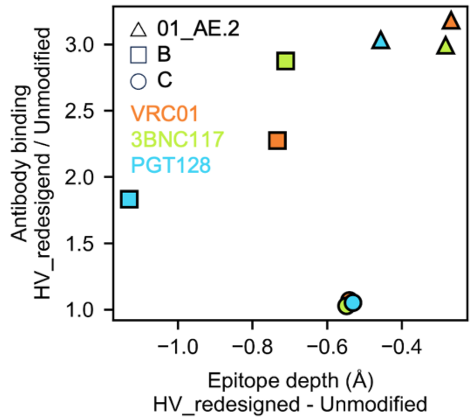


**Fig. S18. Comparison between the improvement of antibody accessibility and the improvement in antibody binding.** The epitope depth corresponds to the mean value of 100 lowest energy models using loops modeled with the KIC loop modeling protocol of Rosetta. Source data for panel (A) are provided in the Source Data file.

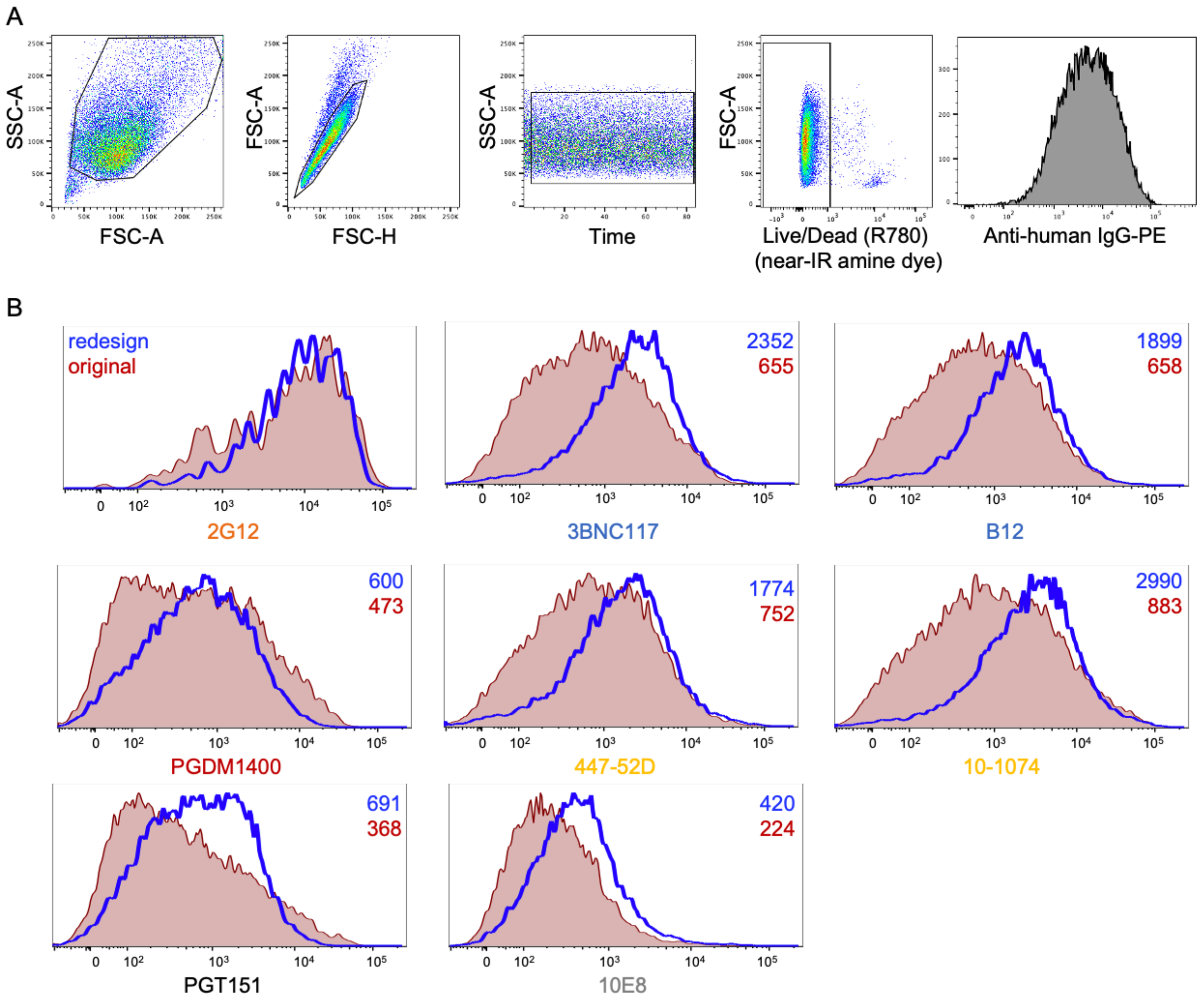


**Fig. S19. BnAb epitope recognition for HIV-1 subtype Env consensus sequences with or without HV loop modifications.** 293T cells transfected with DNA expression constructs were stained with bNAbs and analyzed by flow cytometry. (A) Flow cytometry gating tree applied to mRNA-transfected 293T cells. Sequential gating was applied as shown from left to right. (B) Eight bnAbs were used to compare the cell surface expression of Env consensus sequences with unmodified (red) and redesigned (blue) HV loops. Median fluorescent intensity values are indicated; 2G12 reference staining confirms comparable protein expression.

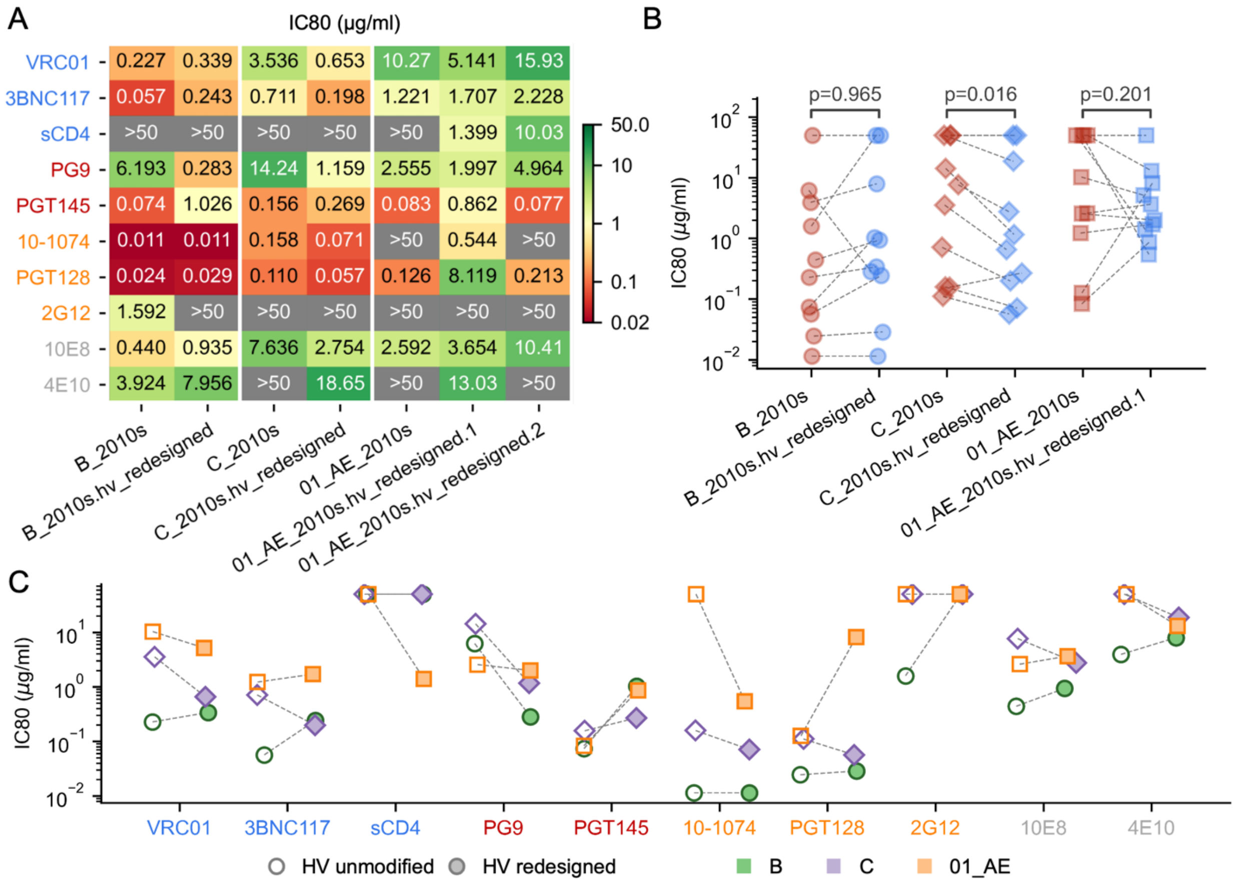


**Fig. S20. Impact of HV loop modifications on the sensitivity of pseudoviruses to ten bnAbs.** (A) Neutralization sensitivity of the consensus Env with or without redesigned HV loops for subtypes B, C and CRF01_AE measured for ten bnAbs that targeted the CD4 binding site (VRC01 and 3BNC117), V2-apex (PG9 and PGT145), glycan supersite (10-1074 and PGT128), MPER (10E8 and 4E10) and mannose-dependent (2G12) epitopes as well as the sCD4. The neutralization sensitivity is reported as IC80 values (μg/ml) with the most sensitive viruses colored in red and the most resistant in gray. Neutralization sensitivity (IC80 values) shown for the unmodified and redesigned HV loops and displayed separately for each Env clade (B, n=10) or each bnAb (C). The increase in sensitivity with the redesigned HV loops is tested for each clade with one-tailed exact Wilcoxon-Pratt signed-rank tests. Only the 01_AE_2010s.hv_redesigned.1 is shown in panels B and C (it has a shorter V1HV than 01_AE_2010s.hv_redesigned.2). Source data are provided as a Source Data file.
